# Relationship between lower urinary tract symptoms and disease activity among women with systemic lupus erythematosus in Korea: a cross-sectional study

**DOI:** 10.7586/jkbns.25.009

**Published:** 2025-02-25

**Authors:** Hyo Jeong Song, Young-Joo Kim, Jinseok Kim

**Affiliations:** 1 College of Nursing, Health and Nursing Research Institute, Jeju National University, Jeju, Korea; 2 Department of Urology, Jeju National University, Jeju, Korea; 3 Department of Rheumatology, Jeju National University, Jeju, Korea

**Keywords:** Lower urinary tract symptoms, Disease, Urinary incontinence, Lupus erythematosus, systemic

## Abstract

**Purpose:**

Bladder involvement in the disease course of systemic lupus erythematosus (SLE) is uncommon; in fact, it has been shown that patients with SLE have lower urinary tract symptoms (LUTS) than the general population. This study aimed to identify LUTS and the relationship between LUTS and disease activity among women with SLE.

**Methods:**

This cross-sectional study used structured self-administered questionnaires. We recruited 110 women with SLE from the outpatient clinic of a university hospital between January and August of 2020. LUTS was assessed using the International Prostate Symptom Score, and disease activity was assessed using the Systemic Lupus Activity Questionnaire.

**Results:**

Fifty-seven women (51.8%) reported urinary incontinence (UI), while 53 (48.2%) reported no UI. The mean LUTS score was 7.75 ± 5.74 (range 0-35). The scores for the seven LUTS components (range 0-5) were as follows: frequency (1.51 ± 1.48), nocturia (1.46 ± 1.07), urgency (1.27 ± 1.35), incomplete emptying (1.09 ± 1.15), intermittency (0.97 ± 1.27), weak stream (0.94 ± 1.04), and straining (0.51 ± 0.91). LUTS were positively correlated with the disease activity of SLE (r = .48, *p* < .001), indicating that higher LUTS scores were associated with severe disease activity.

**Conclusion:**

The prevalence of UI was high, and LUTS (frequency, nocturia, and urgency) were common. Therefore, regular assessment and appropriate management of UI and LUTS among women with SLE are necessary, and it is also essential to keep the disease activity of SLE at a low level due to the positive relationship between disease activity and LUTS.

## INTRODUCTION

Systemic lupus erythematosus (either SLE or lupus) is an autoimmune disease in which circulating autoantibodies and immune complexes invade multiple body organs, including the joints, skin, brain, cardiovascular system, lungs, and kidneys, causing chronic inflammation and tissue damage. Unpredictable flares and remissions characterize SLE through a chronic disease course [1].

Approximately 3.4 million individuals worldwide have been diagnosed with SLE, of whom approximately 3 million are women [2]. Within Asia, the lupus prevalence ranges from 26.5–103 per 100,000 individuals [3]. According to the National Health Insurance database, the annual prevalence of SLE in Korea increased slightly from 21.25/100,000 people in 2005 to 35.45/100,000 people in 2015. From 2005 to 2015, the incidence of SLE among women was 6- to 8-fold higher than that among men [4].

SLE often progresses to urinary tract disease, most commonly nephritis, due to the deposition of immune complexes in the renal glomeruli. Fibrosis and thinning of the bladder wall led to a gradual decrease in bladder capacity and obstructive uropathy symptoms [5]. Although the pathological cause or mechanism of lupus cystitis is not yet clearly understood, histopathological studies suggest a possible role of immune complex-mediated small vessel vasculitis [6]. Lupus cystitis symptoms include lower urinary tract symptoms (LUTS) such as urgency, frequency, dysuria, and nocturia, as well as pain or discomfort in the suprapubic region, and it is known that when it progresses, it causes permanent bladder dysfunction and irreversible renal dysfunction as significant complications [7]. Haarala et al. [5] conducted a study on LUTS patients that showed a higher prevalence rate than the general population; thus, it was reported that autoimmune disorders owing to lupus can affect the bladder. In a study on urinary disorders in women with SLE, Yu et al. [6] showed that frequency, urgency, weak stream, residual urine, and severe LUTS were significantly higher among SLE women than in the healthy control group. In a study on the urinary function of ten patients with SLE, Duran-Barragan et al. [8] showed that most patients had urgency, frequency, nocturia, and bladder pain, and their urodynamic tests showed characteristics such as decreased bladder capacity and decreased bladder sensation. As such, SLE patients experienced a high prevalence of urinary problems, and the degree of symptoms has been observed to be more severe than in the general population. In addition, musculoskeletal damage occurring during SLE disease can damage the pelvic floor muscles and affect the development of LUTS [9]. Still, the prevalence is not yet well known, and awareness of its importance in management is low compared to other symptom management. LUTS are often overlooked but can significantly impact an individual's quality of life [10]. Therefore, regular assessment and examination of urinary tracts in SLE patients can detect LUTS and urinary voiding dysfunction early, which can be an essential opportunity for appropriate care and treatment.

SLE disease activity significantly impacts subsequent organ damage and quality of life. It has been reported that when lupus' low disease activity state is well maintained, it is associated with significant protection from disease relapses, irreversible organ damage, mortality, and deterioration of quality of life [11]. The disease activity of SLE may affect LUTS. Yu et al. [6] also reported that the higher the disease activity, the more serious the urinary symptoms are.

Therefore, this study aimed to identify the prevalence of LUTS among women with SLE and the relationship between SLE disease activity and LUTS.

## METHODS

### 1. Study design

This cross-sectional study was conducted to identify the prevalence of LUTS among women with SLE and to determine the relationship between SLE disease activity and LUTS.

### 2. Participants

The participants of this study were women aged ≥ 18 years who had been diagnosed with SLE and had been visiting the hospital outpatient clinic for > 6 months and receiving treatment regularly. The number of participants was calculated using the G*Power 3.1.9.2 program [12], and the minimum number of participants was 109 based on the two-tailed test for correlation, with a medium effect size of .30, significance level of .05, and power of .90 [13]. After excluding one participant with insufficient questionnaire data, there were 110 participants. The selection criteria for participants were those who understood the purpose of the study and gave written consent to participate.

### 3. Instruments

#### 1) General and disease-related characteristics

General and disease-related characteristics included age, education level, living with spouse, body mass index (BMI, m^2^/kg) using self-reported height and weight, drinking, smoking, alcohol consumption, disease duration, and comorbid disease.

#### 2) LUTS

The definition of LUTS was systematized by the International Continence Society (ICS) in 2002 to include all urinary symptoms and is used worldwide. Urinary incontinence (UI) is included as one of the LUTS to the bladder storage problem of overall LUTS. but it is named UI and LUTS depending on the measurement tool [14].

UI was queried based on the ICS definition (the complaint of involuntary urine leakage). Within this study, UI was operationally defined as a report of involuntary urine leakage less than once a month or more frequently during the three months before data collection. Stress UI (SUI) refers to involuntary urinary leakage during exertion, exercise, laughing, coughing, or sneezing. In contrast, urge UI (UUI) refers to urinary leakage before reaching the toilet accompanied by urgency. Mixed UI (MUI) refers to cases where symptoms of both SUI and UUI were reported [14-16].

LUTS were measured using a Korean translation of the International Prostate Symptom Score instrument [17]. LUTS have seven sub-symptoms: incomplete emptying, frequency, intermittency, urgency, weak stream, hesitancy, and nocturia. The score scale for each sub-symptom is 0 points for ‘never,’ 1 point for ‘rarely (one out of five times),’ 2 points for ‘sometimes (one or two out of five times),’ 3 points for ‘approximately half of the time (two or three out of five times),’ 4 points for ‘more than half of the time (three or four out of five times),’ and 5 points for ‘always.’ Regarding LUTS experienced by the participant in the past three months, each lower symptom score ranges from 0–5 points, and the total LUTS score, consisting of 7, ranges from 0~35 points. The tool's reliability for measuring LUTS in women and men was found to be Cronbach's α = .80 [18], and in this study, the reliability was Cronbach's α = .81.

#### 3) Disease activity

Disease activity was measured using the SLE Disease Activity Questionnaire developed by Karlson et al. [19]. This tool consists of 24 self-report questions about symptoms experienced in the past three months and one question about overall disease activity. This study used a 24-item symptom scale including weight loss, fatigue, fevers, oral ulcers, malar rash, photosensitivity, vasculitis, other rashes, alopecia, lymphadenopathy, dyspnea, chest pain, Raynaud’s phenomenon, abdominal pain, paresthesia, seizures, stroke, memory loss, depression, headaches, myalgias, muscle weakness, arthralgias, and joint swelling. The score for each symptom ranges from 0 (not at all difficult) to a maximum of 3 (severely difficult), and each symptom is weighted according to the scoring method proposed by Karlson et al. [19]. The total score ranges from 0 to 47, with a higher score indicating higher disease activity. The tool's reliability was Cronbach's α = .85 in a large observational cohort study [20], and the tool's reliability was Cronbach's α = .87 in the present study.

### 4. Data collection

This study collected data using a self-report questionnaire from patients who regularly visited Jeju National University Hospital in Jeju City for lupus treatment and management from January to August 2020. First, approval was obtained from the institutional research ethics review committee, and permission was obtained to collect data from institutional officials. The researcher explained this study's purpose and the questionnaire's contents to patients waiting to see an outpatient clinic. If they met the research subject criteria, they received written consent for participation in the study and then administered the self-report questionnaire. The questionnaire took approximately 15 minutes to complete.

### 5. Data analysis

The data was analyzed using the SAS 9.2 (SAS Institute Inc., Cary, NC, USA). According to the characteristics, LUTS were analyzed using independent t-tests and ANOVA. The relationship between LUTS and SLE disease activity was calculated using Pearson correlation.

### 6. Ethical considerations

This study was conducted after an ongoing regular review by the Jeju National Hospital Institutional Review Board (approval number: JEJUNUH 2019-09-010-002). The purpose of the study, procedures, information on anonymity and confidentiality, contact information for the researcher, and the right to refuse participation at any time during the study were clearly stated in the consent form. We explained the purpose and method of the study to the participant sufficiently and then obtained the patient's voluntary consent and signature. The collected data were processed anonymously to protect the participant's identity, and the questionnaire data were stored in a locker accessible only to the researcher to ensure confidentiality. After the study, the researcher stored the data for the period designated by the Research Ethics Review Committee and then destroyed it.

## RESULTS

### 1. Participant characteristics

In this study, 110 women with SLE participated. The mean age was 44.74 ± 14.07 years, with 65.5% under 50 ranging from 18–75 years. 53.6% had a college and an over education level, and 63.6% lived with a spouse. Employment was reported by 58.7%. In addition, 34.9% consumed alcohol, whereas 11.0% were current smokers. The mean BMI was 23.39 ± 4.47 kg/m^2^, with 26.4% of the participants having a 25 kg/m^2^ or higher BMI. The mean disease duration was 157.61 ± 107.83 months; 44.5% of women had 120 months or less. 48.2% had a comorbid condition. The disease activity mean score was 12.84 ± 8.37 (Table 1).

### 2. UI and LUTS

Fifty-seven subjects (51.8%) reported UI. The majority of reported types of UI were SUI (33.6%), MUI (16.4%), followed by UUI (1.8%), and no UI (48.2%). Among women with UI, the prevalence of UI was ‘less than once monthly’ 22.7%, ‘one or several times monthly’ 17.3%, ‘one or several times weekly’ 8.2%, and ‘daily’ 3.6% (Table 2).

The mean score for LUTS among the women was 7.75 ± 5.74. Each LUTS symptom score was as follows: urinary frequency, 1.51; nocturia, 1.46; urgency, 1.27; and incomplete emptying, 1.09 (Table 3).

### 3. LUTS based on participant characteristics

There was no statistically significant difference in LUTS according to the characteristics of women with SLE (Table 4).

### 4. Association between LUTS and disease activity

The relationship between LUTS and disease activity showed a statistically significant correlation (r = .48, *p* < .001); this indicates that the higher the LUTS scores, the more severe the disease activity is (Table 5).

## DISCUSSION

UI is defined by ICS 2002 or International Urogynecological Association/ICS 2010 as the involuntary loss of urine. It includes UUI, SUI, or MUI [21]. Based on the UI definition by ICS, the prevalence of UI in this study was high at 51.8%, and the types of UI were SUI 33.6%, MUI 16.4%, and UUI 1.8%. In the study of Abushamma et al. [22], which targeted 89 women with the same type of rheumatoid arthritis, the prevalence of SUI was high at 40.4% and UUI at 30.3%. When comparing these results with the women in this study, the UUI level was lower. However, since there were very few studies investigating UI, there were limitations in comparing and discussing the results of this UI with those of previous studies. In SLE patients, UI may develop from direct inflammation and damage to the bladder wall and neurogenic bladder dysfunction due to SLE nervous system involvement [7,23]. Furthermore, SLE can affect pelvic floor muscles [24], which may involve stress and other UI and its risk factors. For this reason, we have assessed UI using symptom questionnaires to examine UI and its patterns, revealing a high prevalence of UI among women with SLE in this study. Therefore, it is important to evaluate further diagnostic tests to identify the UI and UI patterns because the cause, management, and proper treatment differ depending on the UI type and severity.

In this study, LUTS of SLE women showed the highest score of frequency, and nocturia and urgency were high, with storage LUTS predominating. This result showed a similar pattern to the LUTS symptoms of the study of Yu et al. [6]. On the other hand, the results of the study by Sakakibara et al. [23], which performed to evaluate LUTS and urodynamic tests on SLE patients with neurological disorders, showed various and severe voiding symptoms such as voiding difficulty, decreased urine flow, increased maximal urethral closure pressure, bladder hypersensitivity, impaired bladder contractility, and impaired bladder-sphincteric coordination.

SLE disease affects various body parts, symptoms vary from patient to patient, and the course of the disease is not constant even in one patient. Therefore, LUTS and voiding dysfunction due to SLE can vary from individual to individual, so a comprehensive symptom assessment focused on individuals is needed to be approached.

Existing literature highlights the complex relationship between disease activity and urinary symptoms [25]. The significant positive correlation between SLE disease activity and LUTS observed in this study indicates that the higher LUTS scores showed severe disease activity. Similar results were reported by Yu et al. [6], who showed that severe disease activity in SLE women had higher LUTS. Additional studies should be conducted to elucidate the mechanism or pathology of bladder involvement, urinary symptoms, or disorders due to SLE.

This study assessed UI and LUTS in women with SLE and found that they experience them at high levels. In addition, by identifying the positive relationship between LUTS and disease activity, it is necessary to develop nursing interventions to maintain the disease activity of SLE at a low level. The limitations of this study include the insufficient number of subjects, including women with SLE who visited a university hospital, and the reliance on subjective symptom questionnaires. In addition, this study was limited to a single area in a city; there are limitations in generalizing the results to the entire population.

## CONCLUSION

Our study showed that the prevalence of UI in women with SLE was high, and LUTS was predominant in the order of frequency, nocturia, and urgency. The more severe the disease activity, the higher the LUTS. Management to maintain a low level of disease activity owing to SLE is emphasized, which can prevent conditions that may trigger or worsen LUTS. Future studies are required to determine how UI and LUTS influence quality of life. In addition, regular examination of urinary function and appropriate management by medical personnel are essential.

## Figures and Tables

**Table 1.** General and Disease-related Characteristics of the Participants (N = 110)

Characteristics	Categories	n (%)	M ± SD
Age (yr)	≤ 50	72 (65.5)	44.74 ± 14.07
	> 50	38 (35.5)	
Educational level	≤ High school	51 (46.4)	
	College and over	59 (53.6)	
Living with spouse	No	40 (36.4)	
	Yes	70 (63.6)	
Employment^†^	No	45 (41.3)	
	Yes	64 (58.7)	
Alcohol intake^†^	No	71 (65.1)	
	Yes	38 (34.9)	
Smoking^†^	No	97 (89.0)	
	Yes	12 (11.0)	
Body mass index (kg/m^2^)	< 25	81 (73.6)	23.39 ± 4.47
	≥ 25	29 (26.4)	
Duration of disease (mon)	≤ 120	49 (44.5)	157.61 ± 107.83
	> 120	61 (55.5)	
Comorbid disease	No	57 (51.8)	
	Yes	53 (48.2)	
Disease activity			12.84 ± 8.37 (Range: 0~47)

M = Mean; SD = Standard deviation.

^†^Total number of participants do not match that of the respondents.

**Table 2.** Distribution of Urinary Incontinence and Type (N = 110)

Characteristics		n (%)
UI		57 (51.8)
	SUI	37 (33.6)
	UUI	2 (1.8)
	MUI	18 (16.4)
Continence		53 (48.2)
UI frequency (Frequency of experiencing UI)	Less than once monthly	25 (22.7)
	One or several times monthly	19 (17.3)
	One or several times weekly	9 (8.2)
	Daily and/or nightly	4 (3.6)
	None	53 (48.2)

UI = Urinary incontinence; SUI = Stress urinary incontinence; UUI = Urge urinary incontinence; MUI = Mixed urinary incontinence.

**Table 3.** The Mean Score for Each Lower Urinary Tract Symptom (N = 110)

Characteristics	Categories	M ± SD	Range
LUTS	Frequency	1.51 ± 1.48	0~5
	Nocturia	1.46 ± 1.07	0~5
	Urgency	1.27 ± 1.35	0~5
	Incomplete emptying	1.09 ± 1.15	0~5
	Intermittency	0.97 ± 1.27	0~5
	Weak stream	0.94 ± 1.04	0~5
	Straining	0.51 ± 0.91	0~5
	Total	7.75 ± 5.74	0~35

M = Mean; SD = Standard deviation; LUTS = Lower urinary tract symptoms.

**Table 4.** Lower Urinary Tract Symptoms by General and Disease-related Characteristics (N = 110)

Characteristics	Categories	LUTS		
		M ± SD	t or F	*p*
Age (yr)	≤ 50	7.64 ± 6.08	−0.29	.772
	> 50	7.97 ± 5.11		
Education level	High school or below	7.96 ± 0.11	0.34	.734
	College or higher other tertiary education	7.58 ± 4.75		
Living with spouse	No	7.08 ± 5.06	−0.94	.350
	Yes	8.14 ± 6.09		
Employment	No	7.40 ± 5.57	0.63	.527
	Yes	8.11 ± 6.74		
Alcohol intake	No	8.13 ± 6.67	0.77	.442
	Yes	7.24 ± 5.66		
Smoking	No	7.30 ± 5.12	−1.88	.085
	Yes	12.00 ± 8.50		
Body mass index (㎏/m^2^)	< 25	7.73 ± 5.97	−0.08	.937
	≥ 25	7.83 ± 5.13		
Duration of disease (mon)	≤ 120	7.29 ± 5.51	−0.77	.445
	> 120	8.13 ± 6.76		
Comorbid diseases	No	6.91 ± 5.73	−1.58	.117
	Yes	8.66 ± 6.79		

M = Mean; SD = Standard deviation; LUTS = Lower urinary tract symptoms.

**Table 5.** Correlation between Lower Urinary Tract Symptoms and Disease Activity

Variables	Disease activity
	r (*p*)
LUTS	.48 (< .001)

LUTS = Lower urinary tract symptoms.

## Data Availability

The data that support the findings of this study are available from the corresponding author upon reasonable request.
